# Enhanced Electro-Optical Properties and Peel Strength of Epoxy-Based Polymer-Stabilized Liquid Crystal Films Enabled by Rapid Cationic Polymerization and Polymer-Network Morphology Regulation

**DOI:** 10.3390/ma18071505

**Published:** 2025-03-27

**Authors:** Zhexu Song, Guangyang Shang, Yingjie Shi, Meiqi Yang, Tianfu Zhou, Meina Yu, Yanzi Gao, Cheng Zou

**Affiliations:** Beijing Advanced Innovation Center for Materials Genome Engineering, Institute for Advanced Materials and Technology, University of Science and Technology Beijing, Beijing 100083, China; songzhexu1108@163.com (Z.S.); shangguangyang@126.com (G.S.); syj0625@163.com (Y.S.); yangmeiqi32@163.com (M.Y.); 18579178779@163.com (T.Z.); yumeina@ustb.edu.cn (M.Y.); gaoyanzi@ustb.edu.cn (Y.G.)

**Keywords:** PSLC films, epoxy, electro-optical properties, mechanical properties, cationic polymerization

## Abstract

Polymer-stabilized liquid crystal (PSLC) dimming film has attracted widespread attention due to its normally transparent state, energy-saving capability, and excellent electro-optical performance, which has promising applications in smart cars and building windows. However, achieving good electro-optical performance and high peel strength simultaneously still remains challenging. In this study, a PSLC film based on monoepoxy and diepoxy monomers was prepared through rapid cationic polymerization, showing low driving voltages and high peel strength simultaneously. The influence of the content and composition of two epoxy monomers on the microstructures, mechanical properties, and electro-optical performance of the PSLC films were systematically studied. The polymer morphology of PSLC could be effectively modulated by doping monoepoxy monomers. The PSLC film with total monomer content of ≤15 wt% showed enhanced electro-optical properties and peel strength when doping monoepoxy monomers due to the lateral polymer in the networks and denser polymer on the substrate. When the ratio of E6M to E6PM was 12:3, compared with pure E6M, the threshold voltage decreased from 18.2 V to 12.6 V, and the peel strength increased from 62.53 kPa to 136.37 kPa. These PSLC films can adapt to the requirements of different application scenarios by changing the content and proportion of two epoxy monomers, and the strategy has good prospects in the actual production and application of PSLC films.

## 1. Introduction

In today’s era, with rapid socioeconomic and technological development, environmental protection and energy utilization issues have attracted much attention [1]. Green and low-carbon development has become the theme of the times, and the development and utilization of renewable energy such as electricity and nuclear energy are becoming increasingly important [2]. People are constantly seeking diverse approaches to enhance energy utilization efficiency and identify methods to cut down on energy consumption. In recent years, electrically controlled light-modulating films have witnessed growing application in areas such as smart windows in building exterior walls and automotive glass. These films can achieve a transition between transparent and light-scattering states, for example, by changing the refractive index matching degree between the liquid crystal and polymer matrix inside through methods such as applying electric fields [3,4]. Current electrically controllable light-modulating films predominantly employ three distinct materials and mechanisms: liquid crystal (LC)-based, electrochromic (EC), and suspended particle (SP) systems. LC-based films rely on LC molecules dispersed within a polymer matrix, where an applied electric field induces molecular reorientation. This field-driven alignment modulation enables reversible optical transitions between light-scattering (opaque) and index-matched (transparent) states through controlled director configuration changes. EC-type devices operate through electric-field-driven ion migration (e.g., Li⁺) between electrochromic layers (e.g., WO_3_) and ion-storage counterparts, inducing reversible redox reactions that alter the material’s light-absorption properties [5,6,7]. SP-type films rely on nano-/microparticles suspended in dielectric fluids, achieving opacity via random particle distribution at the null field and transparency through field-aligned particle orientation [8,9]. Compared with their EC and SP counterparts, LC smart films demonstrate superior optical performance, lower power consumption, and extended operational lifetime, rendering them preferable for architectural and vehicular applications [10,11].

According to their structures, electrically controlled liquid crystal (LC) light-modulating films can be categorized into polymer-dispersed liquid crystal (PDLC) film and polymer-stabilized liquid crystal (PSLC) films. The prevailing electrically controllable smart films in current industrial production and daily application are PDLC films. These films exhibit a light-scattering opaque state under zero-field conditions and transform into a transparent state when energized with an electric field [12]. PSLC films, conversely, maintain transparency normally and transform to opacity under an electric field, demonstrating inherent advantages in smart window applications in cars and buildings, thereby presenting transformative potential for these energy-sensitive applications [13,14,15,16,17]. PSLC films exhibit stable optical states governed by field-dependent molecular ordering [18]. In the voltage-off state, spontaneous homeotropic alignment of mesogens within the polymer matrix enables light transmission, maintaining transparency via refractive index matching. Upon applied voltage, field-aligned planar orientation induces birefringence mismatch, generating dynamic scattering [19]. Contemporary PSLC films predominantly employ acrylate-based polymer matrices. UV-initiated free radical polymerization induces the formation of intricate three-dimensional polymer networks within the film matrix. However, the elevated crosslinking density inherent to acrylate-based systems engenders a high elastic modulus coupled with low fracture toughness, resulting in compromised mechanical robustness of PSLC films [20]. This intrinsic brittleness manifests as stress-induced cracking during dynamic loading, particularly under cyclic bending tests and tensile deformation.

Because of the disadvantages of acrylic esters, epoxy resin has also been introduced as the polymer matrix for PSLC films. Epoxy resins, characterized by exceptional corrosion resistance [21,22], superior adhesion properties [23,24], and outstanding mechanical robustness, have been extensively utilized as encapsulation materials in semiconductor packaging and high-performance protective coatings [25,26]. Their crosslinked network architecture enables effective liquid crystal droplet confinement in liquid crystal/polymer composite systems. Shen [27] investigated the correlation between the network morphology and electro-optical properties of epoxy-based PSLC films. The results indicated that increases in liquid crystalline epoxide content, UV intensity, and initiator content led to an increase in the density of the polymer network and a decrease in the size of the voids between polymer frameworks, thereby enhancing the stabilizing effect of the polymer network on the mesogenic molecules. Chen et al. [28] fabricated PSLC films through UV curing of a composite-containing negative-type liquid crystal, diepoxy monomer (E6M), and photoinitiator (6976). The optimized formulation (E6M = 10 wt%) demonstrated enhanced mechanical properties but required a prolonged curing duration (120 min) due to restricted epoxy conversion efficiency. Excessive E6M loading (>12 wt%) caused complete loss of electro-optic response. Wu [29] enhanced the structural stability by introducing polymer walls into epoxy-resin-based PSLC films. Moreover, by providing a multifunctional surface in the polymer-wall-stabilized liquid crystal (PWSLC) system and rationally modifying the microstructure as well as controlling the silane components of the multifunctional surface, both the electro-optical and mechanical properties were improved simultaneously. Shi [30] constructed a vertically oriented layer using a silane coupling agent via a two-step self-assembly process, thereby enhancing the mechanical strength of PSLC through covalent bonding within the polymer network. Although the peel strength of PSLC films has been greatly improved, it is still far from the requirements for practical applications. In practical applications, PSLC films need not only to maintain their excellent electro-optical properties but to have high peel strength, to maintain their structural integrity in the face of various complex environmental factors, and to have excellent antiaging performance.

In this study, a high-performance PSLC film was attained via UV-light-induced rapid cationic polymerization of epoxy monomers, facilitated by dual photoinitiators 1173 and 1176. This formula reduced the polymerization time from 2 h to 5 min, which is highly beneficial for the production of large-area flexible film. In addition, the study demonstrates a monomer engineering strategy for developing low-voltage PSLC films through controlled copolymerization of diepoxy monomer E6M and monoepoxy monomer E6PM. The effects of the content and composition of these two epoxy monomers on the microstructures, mechanical properties, and electro-optical performance of the PSLC films were systematically studied. Doping E6PM into an E6M polymer matrix when the total monomer content ≤ 15 wt% reduced the driving voltage and increased the peel strength of the resulting PSLC film. This study provides an efficient strategy for the fabrication of PSLC films and a new perspective for the optimization of their performance.

## 2. Experimental Section

### 2.1. Materials

**Figure 1:**
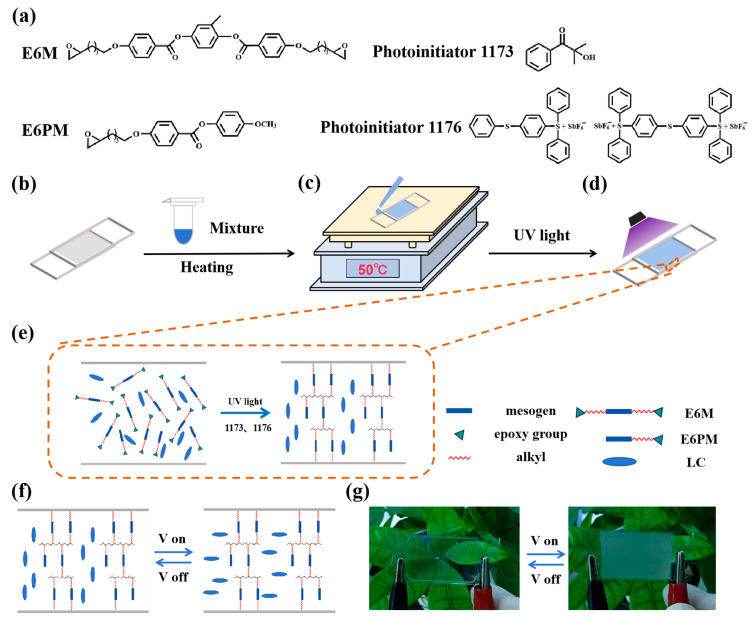
(**a**) Molecular structures of E6M, E6PM, photoinitiator 1173, and photoinitiator 1176 used in the experiments; (**b**–**d**) schematic diagram showing the preparation process of the PSLC films; (**e**) schematic diagram of the polymer network topology; (**f**) schematic showing the molecular arrangement when the sample was powered on and off; (**g**) photos of the PSLC film when powered on and off.

The nematic liquid crystal with negative dielectric anisotropy used in the experiments was GXV-7822-180 (T_NI_ = 380.15 K, Δn = 0.18, n_o_ = 1.504, n_e_ = 1.684), purchased from Yantai Xianhua Technology Group Co., Ltd. (Yantai, China). The monomers used in the experiments were E6M (4′-epoxycyclohexylmethyl 3,4-epoxy cyclohexane carboxylate) and E6PM (4-methoxyphenyl4-(5,6-epoxyhexoxy)benzoate), purchased from Nanjing Shuxin Science and Technology Co., Ltd. (Nanjing, China). Photoinitiator 1173 and photoinitiator 1176 were purchased from Shanghai Aladdin Biochemical Technology Co., LTD. (Shanghai, China). The molecular structures of the monomers and photoinitiators used in this study are shown in Figure 1a.

### 2.2. Sample Preparation and Working Mechanism

For epoxy monomers, light-polymerization requires high free energy and the use of cationic initiators, unlike acrylic-based polymers, which require free-radical photoinitiators to initiate polymerization. In this study, photoinitiator 1173 decomposed into phenylcarbonyl and 2-hydroxypropan-2-yl radicals under ultraviolet light irradiation and was oxidized by photoinitiator 1176 to form the phenylcarbonyl carbocation. Subsequently, the epoxy monomers could be polymerized under the reaction of the carbocation. The sample preparation process is schematically shown in Figure 1. First, the negative liquid crystal GXV-7822-180, diepoxy monomer E6M, monoepoxy monomer E6PM, photoinitiator 1173, and photoinitiator 1176 were mixed in proportion in a centrifuge tube. The centrifuge tube was subsequently placed on a shaker and subjected to vigorous shaking. During the shaking period, a hair dryer was employed to blow on and heat the mixture evenly. Subsequently, the liquid mixture was carefully dispensed onto a conductive glass substrate coated with indium tin oxide (ITO) using a pipette. The liquid was then drawn into the gap between the two conductive substrates via capillary action. Additionally, a vertically oriented polyimide (PI) layer was deposited on the ITO surface. The thickness of the gap between the two conductive substrates was maintained at 20 μm, controlled by microbeads or adhesive spacer. The cell was placed on a heating stage at a constant temperature of 50 °C so that the liquid mixture was injected in at a slightly faster and more uniform rate. After the mixture completely filled the entire conductive substrate, it was moved into a UV light box (365 nm, irradiation size 100 mm × 100 mm, Yuntong Technology (Shanghai, China)) for UV-induced cationic polymerization. The polymer topology of the sample is schematically shown in Figure 1e. The E6PM served as a pendent mesogenic unit to reduce the crosslinking density of the polymer networks. As depicted in Figure 1f, the molecular arrangement in the sample without an applied electric field was homeotropic, and a transparent film sample is shown in Figure 1g. When an external electric field was applied to the sample, the liquid crystals rotated, and the sample turned opaque, as shown in Figure 1g.

### 2.3. Characterization

The instrument used for electro-optical performance testing in this experiment was the Liquid Crystal Comprehensive Parameter Tester LCT-P1000 (Changchun North Liquid Crystal Engineering Research and Development Center Co., Ltd., Changchun, China). The incident light source used for the test was a halogen laser (λ = 560 nm) with an external electric field driving waveform of square wave frame inversion (100 Hz). The photodiode detector received the signal passing through the sample, and the collection angle was controlled at ±2°. Before testing the sample, the transmittance of the air was measured to normalize the transmittance of the samples. The ballistic light measurement method was used to measure transmittance when the voltage was turned on. The polymer network morphology of the samples was measured using a scanning electron microscope (SEM, HITACHI S-4800, Tokyo, Japan). Before observation, the samples were cut into pieces and soaked in cyclohexane for 4 days. The cyclohexane was replaced daily to ensure that the liquid crystal small molecules in the samples were completely removed. Then, the samples were dried in a vacuum oven at 60 °C for 12 h. Finally, the surface of the samples was sprayed with gold, and the treated samples were observed by SEM.

## 3. Results and Discussion

First, the effect of the monoepoxy monomer on the performance of PSLC films was investigated on the samples when the total content of the diepoxy monomer E6M and the monoepoxy monomer E6PM was 15 wt%. The composition and polymerization conditions for these samples are shown in Table 1.

**Figure 2:**
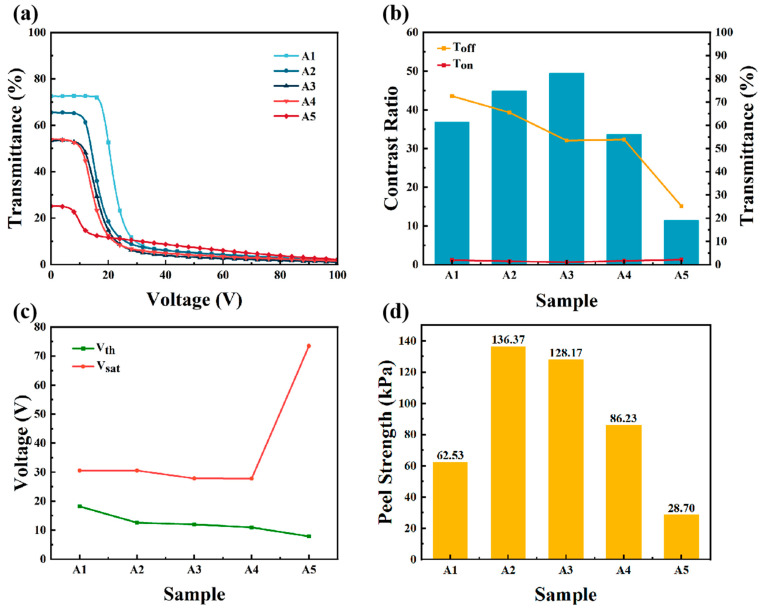
Electro-optical and mechanical performance when the total content of E6M and E6PM was 15 wt%: (**a**) transmittance–voltage curves, (**b**) contrast ratios and transmittance, (**c**) driving voltage, and (**d**) peel strength of Samples A1–A5.

**Figure 3:**
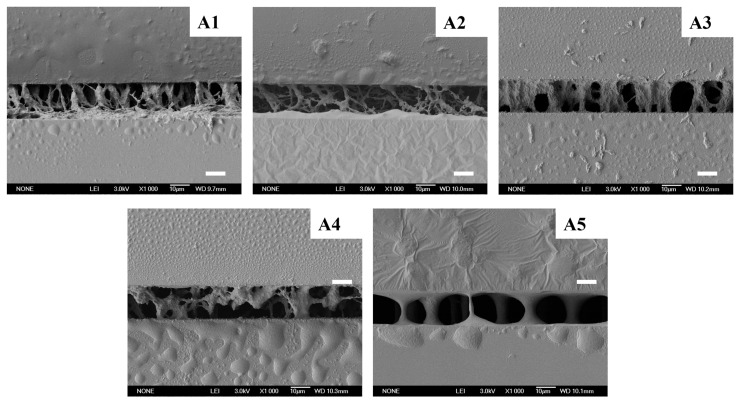
SEM images of Samples A1–A5 (scale bar: 10 μm).

The electro-optical properties and peel strength of Samples A1–A5 were characterized and are shown in Figure 2. The polymer morphology of these samples were shown in Figure 3. As shown in Figure 2b, when the total content of E6M and E6PM was 15 wt%, the contrast showed a trend of first increasing and then decreasing with increasing EP6M content E6PM. This trend was associated with the polymer morphology shown in SEM images (Figure 3). When the ratio of E6M to E6PM was 12:3, the polymer fibers between the two conductive substrates became sparse and exhibited a certain degree of lateral growth (as shown in Figure 3 (A2)). However, these lateral polymer networks induced the liquid crystal molecules to exhibit a certain degree of lateral arrangement, resulting in a decrease in initial transmittance [31]. When Sample A2 was powered on, the liquid crystal molecules were aligned well by the electric field, leading to a significant decrease in transmittance compared with pure E6M, resulting in an improved contrast ratio (contrast ratio = Toff/Ton). When the ratio of E6M to E6PM was 9:6, the polymer fibers became sparser, and the domain density decreased (as shown in Figure 3 (A3)). When Sample A3 was powered off, the orientation of the liquid crystal molecules became disordered, resulting in a further decrease in initial transmittance. Conversely, when Sample A3 was powered on, the liquid crystal molecules aligned more readily with the electric field, achieving more scattering interfaces. Consequently, when charged, the transmittance decreased significantly, and the contrast ratio slightly improved. When the ratio of E6M to E6PM was 6:9, the polymer fibers were sparse, and there were polymer fibers that could not be in contact with both conductive substrates at the same time. Therefore, when the initial transmittance remained unchanged, the scattering interfaces at the On state were reduced because of the sparser polymer networks [32]. This led to higher transmittance in the On state and a reduced contrast ratio.

As shown in Figure 2c, the driving voltage gradually decreased with increasing E6PM content and decreasing E6M content in the composites. According to the SEM images A1–A4 in Figure 3, as the ratio of E6M to E6PM decreased, the polymer fiber network between the two conductive substrates gradually became sparser, and the anchoring effect of the polymer network on the liquid crystal small molecules gradually decreased. When an electric field was applied, the liquid crystal molecules were more easily oriented with the electric field, and the driving voltage became lower. Consequently, the threshold voltage (Vth) and saturation voltage (Vsat) gradually decreased. For Sample A5, when the ratio of E6M to E6PM was 3:12, the density of the polymer fiber network was quite low, and the anchoring effect of polymer fibers on the liquid crystal molecules was poor. The threshold voltage required for liquid crystal molecules to align with the electric field was small. However, it required higher saturation voltage (Vsat) to achieve a sufficient transmittance decrease, as the scattering interfaces were much fewer than in the other samples because of the much sparser polymer networks [33].

As shown in Figure 2d, as the ratio of E6M to E6PM decreased, the mechanical properties of the samples showed a trend of first increasing and then decreasing. This phenomenon could be explained by the polymer morphology change in Samples A1–A5 depicted in Figure 3. When the content of the monoepoxy monomer E6PM was low, increasing the E6PM content enhanced the lateral connection among the polymer networks and the adhesion of the polymer network to the glass substrate surface. Additionally, the polymer network density remained relatively high, leading to improved peel strength. As the E6PM content continued to increase, although there was more adhesion on the interface, the network became very sparse, resulting in a decrease in overall peel strength. This was attributed to the presence of a large number of hydroxyl groups (-OH) on glass surfaces [34]. These hydroxyl groups give the glass surface a certain polarity and can form various interactions with other substances, including hydrogen bonds. Because of the presence of only one reaction site, the polymer chain structure formed by monoepoxy monomers is relatively simple, with the molecular chain ends being relatively unconstrained [35]. Following the epoxy-ring-opening polymerization, the newly generated hydroxyl groups (-OH) can form hydrogen bonds with the hydroxyl groups present on the glass surface. Moreover, the chain structure of monofunctional epoxy polymers is relatively flexible, allowing the functional groups at the chain ends to approach the hydroxyl groups on the glass surface, thereby forming hydrogen bonds. The presence of these hydrogen bonds enabled the polymer to stably adsorb onto the glass interface [36].

To further confirm this conclusion, the effect of the monoepoxy monomer on the performance of PSLC films was investigated on the samples when the total content of the diepoxy monomer E6M and the monoepoxy monomer E6PM was 10 wt%. The compositions and polymerization conditions of Samples B1–B5 are shown in Table 2.

**Figure 4:**
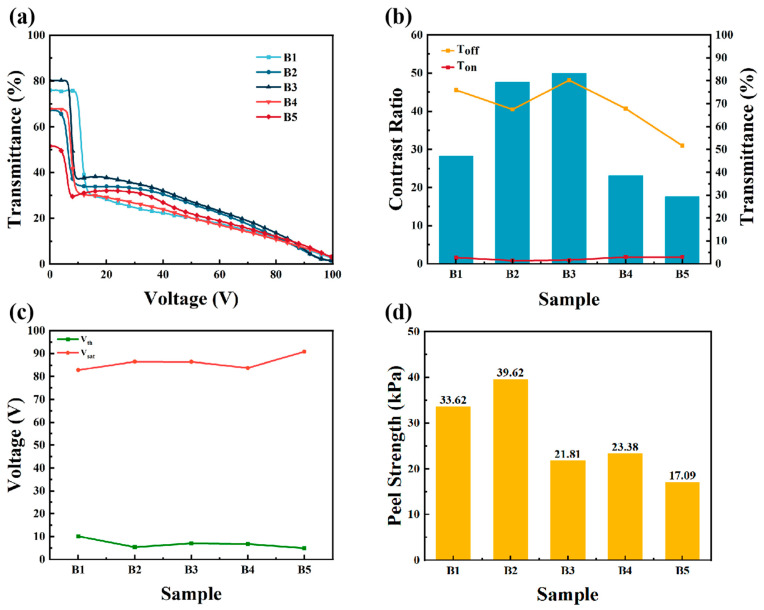
Electro-optical performance and mechanical properties of PSLC samples when the total content of E6M and E6PM was 10 wt%: (**a**) T-V curves, (**b**) contrast ratios and transmittance, (**c**) driving voltage, and (**d**) peel strength of Samples B1–B5.

As shown in Figure 4a,b, when the total content of E6M and E6PM in the mixture was 10 wt%, the contrast ratio showed a trend of first increasing and then decreasing with increasing E6PM content. The trend was similar to that for the samples in Group A. Furthermore, the initial transmittance of most of the samples was lower than in samples with pure E6M. This indicates that the doping of E6PM might induce the growth of some lateral polymer networks, affecting the initial uniform alignment of the liquid crystals. The threshold voltage decreased with increasing E6PM content. However, because the total monomer content was lower, the polymer fibers became sparse, leading to a much higher saturation voltage in Group B than in Group A.

As shown in Figure 4d, the mechanical properties of the sample initially increased and then decreased with increasing E6PM content. This was because when the ratio of E6M to E6PM was 8:2, the polymer fibers between the two conductive substrates developed small, lateral branches, which induced a certain degree of lateral arrangement in the liquid crystal molecules. The mechanical properties were improved to a certain extent when stretched laterally. However, with further increases in E6PM content, the much sparser polymer networks led to deteriorated mechanical properties in the PSLC films. The enhancement effect of peel strength via E6PM doping still worked when the total content of the monomers was 10 wt%.

Increasing the polymer content is an effective way to increase the processability and mechanical properties of PSLC films. Therefore, a further study was carried out on the effect of monomer composition on the microstructures, electro-optical performance, and peel strength of PSLC films when the total content of epoxy monomers was 20 wt%. The compositions and polymerization conditions of Samples C1–C5 are shown in Table 3.

**Figure 5:**
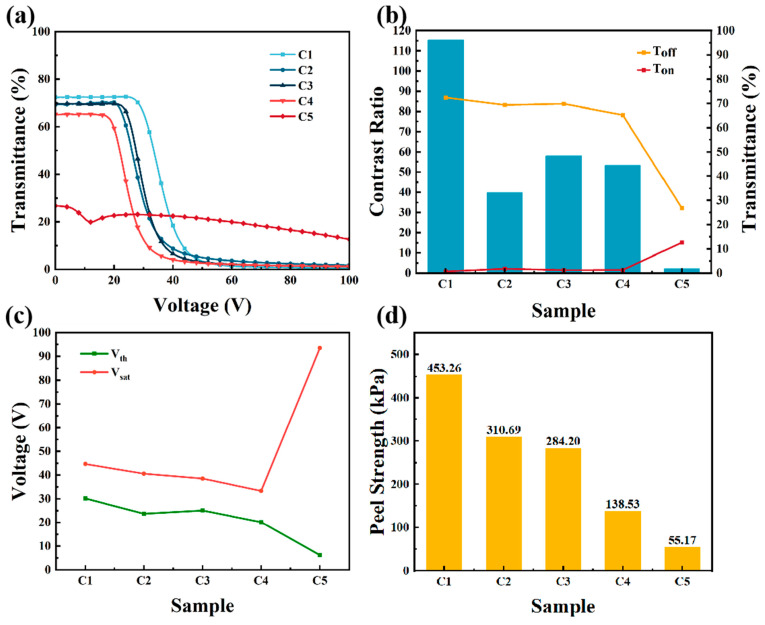
Electro-optical performance and mechanical properties of PSLC samples when the total content of E6M and E6PM was 20 wt%: (**a**) T-V curves, (**b**) contrast ratios and transmittance, (**c**) driving voltage, and (**d**) peel strength of Samples C1–C5.

**Figure 6:**
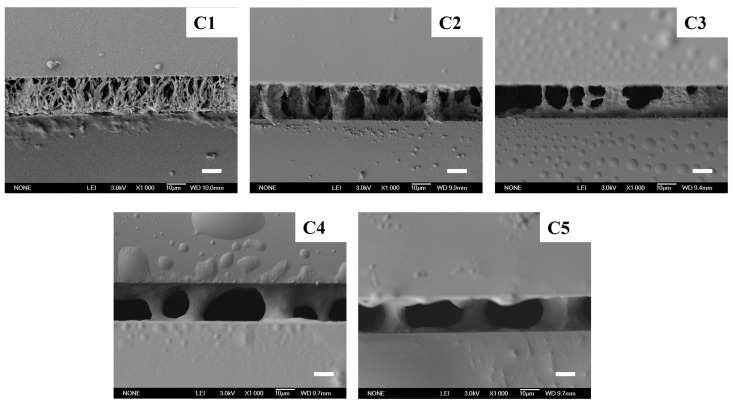
SEM images of Samples C1–C5 (scale bar: 10 μm).

As shown in Figure 5, when the total content of E6M and E6PM was 20 wt%, the contrast ratio of Samples C2–C5 decreased greatly compared with Sample C1. As shown in the SEM images in Figure 6, for Samples C1, C2, and C3, with the addition of E6PM, the polymer fibers between the two conductive substrates gradually became sparse, and the anchoring effect of the polymer network on the liquid crystal molecules gradually deteriorated. In the initial state, the orientation of the liquid crystal molecules was more disordered, so the transmittance gradually decreased. Upon the application of an electric field, the liquid crystal molecules were more likely to orient with the electric field, and the required voltage to achieve the required transmittance change decreased, as confirmed in Figure 5c. When the ratio of E6M to E6PM was 8:12, compared with Sample C3, the initial transmittance of the C4 sample remained essentially unchanged. However, because of the sparser polymer fiber network between the two conductive substrates, the anchoring effect of the polymer network on the liquid crystal molecules further deteriorated. Therefore, when an electric field was applied, the transmittance in the On state increased, and the contrast ratio decreased. The domain density of the polymer fiber networks in Sample C5 was further reduced, and there was an increase in free and disordered liquid crystal molecules, resulting in a decrease in initial transmittance. Therefore, as the transmittance in the On state increased, the contrast ratio decreased, the threshold voltage decreased, and the saturation voltage increased.

As shown in Figure 5d, with increasing E6PM in the polymer, the mechanical properties of the samples in Group C gradually decreased, unlike the previous trends. This trend was attributed to the polymer fiber network between the two conductive substrates abruptly becoming sparse with doping of E6PM, as depicted in Figure 6(C2). This decrease in polymer network density greatly influenced the peel strength of the PSLC films. With further increases in E6PM content, the domain density further decreased, and the tensile performance deteriorated. Interestingly, the peel strength of Samples C1–C4 was still higher than that of the best sample in Group A. Nonetheless, the samples in Group A had much lower driving voltages than the samples in Group C. These different samples could meet the requirements of different actual application scenarios.

In Table 4, we compare the saturation voltage and peel strength of PSLC films in this work with those of recently reported PSLC films. The results indicate that the PSLC film prepared in this work had relatively low saturation voltage and maximal mechanical strength.

## 4. Conclusions

In summary, a PSLC film with enhanced electro-optical performance and peel strength was attained via rapid cationic polymerization of diepoxy and monoepoxy monomers. The doping of monoepoxy monomers improved both the electro-optical and mechanical performance when the total content of monomers was below 15 wt%. The peel strength could reach up to 136.37 kPa, much higher than that of traditional PSLC films, while a Vsat was maintained of about 30 V. In this situation, via monoepoxy monomer doping, the amount of polymer network adherence between the two substrates increased, while the density of the polymer network did not decrease much, so the peel strength increased. As the content of monofunctional monomers continued to increase, although there was more adhesion on the glass interface, the network became very sparse, so the overall peel strength decreased, and the driving voltages of the film showed a decreasing trend. When the total content of E6M and E6PM was 20 wt%, the doping of E6PM decreased both the peel strength and the driving voltages. However, the mechanical properties of the film were excellent, with a peel strength dozens to hundreds times that of previously reported PSLC films with 4 wt% monomer. By changing the content of E6M and E6PM, PSLC films can strike a balance between electro-optical and mechanical performance. This strategy has good prospects in the actual production and application of PSLC films. The performance requirements faced by PSLC films vary in different application scenarios. For example, low-driving-voltage films are required on automotive glass to reduce energy consumption. In building glass curtains, high-peel-strength PSLC films are required to ensure durability. In view of this, we can flexibly adjust the content and proportion of monomers in PSLC films to accurately adapt to specific needs in different scenarios. Most importantly, this strategy reduces the cationic polymerization time from hours to minutes, which is highly beneficial for large-area flexible film production.

## Figures and Tables

**Table 1:** Composition and polymerization conditions for samples of Group A.

Sample No.	Component Content (wt%) and Polymerization Conditions						
	E6M	E6PM	GXV-7822-180	Photoinitiator 1173	Photoinitiator 1176	UV Intensity (mW/cm^2^)	Time (s)
A1	15	0	81	2	2	180	300
A2	12	3	81	2	2	180	300
A3	9	6	81	2	2	180	300
A4	6	9	81	2	2	180	300
A5	3	12	81	2	2	180	300

**Table 2:** Composition and polymerization conditions for samples of Group B.

Sample No.	Component Content (wt%) and Polymerization Conditions						
	E6M	E6PM	GXV-7822-180	Photoinitiator 1173	Photoinitiator 1176	UV Intensity (mW/cm^2^)	Time (s)
B1	10	0	86	2	2	140	300
B2	8	2	86	2	2	140	300
B3	6	4	86	2	2	140	300
B4	4	6	86	2	2	140	300
B5	2	8	86	2	2	140	300

**Table 3:** Composition and polymerization conditions for samples in Group C.

Sample No.	Component Content (wt%) and Polymerization Conditions						
	E6M	E6PM	GXV-7822-180	Photoinitiator 1173	Photoinitiator 1176	UV Intensity (mW/cm^2^)	Time (s)
C1	20	0	76	2	2	240	300
C2	16	4	76	2	2	240	300
C3	12	8	76	2	2	240	300
C4	8	12	76	2	2	240	300
C5	4	16	76	2	2	240	300

**Table 4:** Comparison of saturation voltage and peel strength reported in this work and previous studies.

Reference	Sample Type	Monomer Content (wt%)	Peel Strength (KPa)	V_sat_ (V)
This Work	PSLC	15	136.37	30.56
Ref. [16]	PSLC	16	Not mentioned	>30
Ref. [28]	PSLC	10	Not mentioned	>60
Ref. [29]	PSLC	10	7.5	40.2
Ref. [30]	PSLC	20	71.16	<25
Ref. [37]	PSLC	4	1.6	Not mentioned
Ref. [37]	PSLC	20	49.7	39.12

## Data Availability

The original contributions presented in this study are included in the article. Further inquiries can be directed to the corresponding author.
